# Intestinal Mucosal Barrier Function Restoration in Mice by Maize Diet Containing Enriched Flavan-4-Ols

**DOI:** 10.3390/nu12040896

**Published:** 2020-03-25

**Authors:** Binning Wu, Rohil Bhatnagar, Vijaya V. Indukuri, Shara Chopra, Kylie March, Nina Cordero, Surinder Chopra, Lavanya Reddivari

**Affiliations:** 1 Department of Plant Science, The Pennsylvania State University, University Park, PA 16802, USA; bxw47@psu.edu (B.W.); rohilsahaibhatnagar@gmail.com (R.B.); 2 Interdisciplinary Graduate Program in Plant Biology, The Pennsylvania State University, University Park, PA 16802, USA; 3 Department of Food Science, Purdue University, West Lafayette, IN 47907, USA; 4 Department of Food Science, The Pennsylvania State University, University Park, PA 16802, USA; vijay.pennstate@gmail.com; 5 Department of Biology, The Pennsylvania State University, University Park, PA 16802, USA; soc5439@psu.edu; 6 Department of Veterinary and Biomedical Sciences, The Pennsylvania State University, University Park, PA 16802, USA; kylie.march1@gmail.com; 7 Department of Biochemistry and Molecular Biology, The Pennsylvania State University, University Park, PA 16802, USA; nrcordero52@gmail.com

**Keywords:** maize, flavonoid, flavan-4-ol, carboxymethylcellulose, colonic inflammation

## Abstract

Inflammatory bowel disease (IBD), a chronic intestinal inflammatory condition, awaits safe and effective preventive strategies. Naturally occurring flavonoid compounds are promising therapeutic candidates against IBD due to their great antioxidant potential and ability to reduce inflammation and improve immune signaling mediators in the gut. In this study, we utilized two maize near-isogenic lines flavan-4-ols-containing *P1-rr* (F+) and flavan-4-ols-lacking *p1-ww* (F−) to investigate the anti-inflammatory property of flavan-4-ols against carboxymethylcellulose (CMC)-induced low-grade colonic inflammation. C57BL/6 mice were exposed to either 1% CMC (*w*/*v*) or water for a total of 15 weeks. After week six, mice on CMC treatment were divided into four groups. One group continued on the control diet. The second and third groups were supplemented with F+ at 15% or 25% (*w*/*w*). The fourth group received diet supplemented with F− at 15%. Here we report that mice consuming F+(15) and F+(25) alleviated CMC-induced increase in epididymal fat-pad, colon histology score, pro-inflammatory cytokine interleukin 6 expression and intestinal permeability compared to mice fed with control diet and F−(15). F+(15) and F+(25) significantly enhanced mucus thickness in CMC exposed mice (*p* < 0.05). These data collectively demonstrated the protective effect of flavan-4-ol against colonic inflammation by restoring intestinal barrier function and provide a rationale to breed for flavan-4-ols enriched cultivars for better dietary benefits.

## 1. Introduction

Inflammatory bowel diseases (IBD), such as Crohn’s disease and ulcerative colitis, are characterized by chronic intestinal inflammation and epithelium damage. IBD not only negatively impacts human gut health but is also associated with other metabolic disorders such as adiposity and colon carcinogenesis [1]. Though the etiology of IBD has not been fully clarified, recent studies have identified various genetic and environmental factors that are closely associated with IBD pathogenesis. Diet is among the most important of these factors with a profound impact on an individual’s health. For example, the westernized diet characterized by high fat, high sugar, high meat, and low plant fiber consumption has been implicated as a risk factor for IBD by several studies [2,3]. Dietary emulsifiers, which are detergent-like molecules used for stabilizing and texturizing purposes in the food industry, are an inherent part of the western diet. Carboxymethylcellulose (CMC) is a commonly used emulsifier and can be found in all kinds of processed foods such as ice creams, ketchup, gluten-free foods, and confectionery products. Moreover, CMC is also a compositional ingredient in non-food sources like toothpaste, mouthwash, laxatives, and diet pills. CMC consumption has been previously reported to alter gut microbiota localization, promote the massive bacterial overgrowth of jejunum and ileum in genetically susceptible IL10^-/-^ mice and induce low-grade colonic inflammation, IBD and metabolic syndrome in wild-type mice [4,5].

The majority of standard IBD therapies using pharmaceutical agents such as aminosalicylates, immunosuppressants, biologicals or combined therapy, though they have been shown to potently alleviate the symptoms in some cases, are not devoid of deleterious side effects [6]. To develop novel and effective therapy against IBD, researchers have focused their attention on plant secondary metabolites such as phenolics. Phenolic compounds are well known for their antioxidant property. Furthermore, accumulating evidence has shown that polyphenols play a role in interfering with the inflammatory signaling pathway to modulate immune responses in the gut [7,8].

Flavonoid compounds are a large subfamily of phenolic compounds abundant in many of the fruits and vegetables that are essential constituents of our daily diet. Substantial evidence has demonstrated the properties of isolated flavonoids or flavonoid-rich fractions as potent anti-inflammatory agents as well as their protective effects against chronic diseases including IBD [9,10,11,12]. Maize (*Zea mays L*.) is one of the most important cereal crops all over the globe, colored maize cultivars rich in flavonoid and carotenoid compounds are thought to confer better health benefit than conventional maize. Specifically, flavan-4-ols such as luteoferol and apiferol, are precursors of phlobaphenes, reddish-brown pigments synthesized through the phenylpropanoid pathway. Flavan-4-ol biosynthesis requires an R2R3-MYB transcription factor *pericarp color1* (*p1*). Maize plants carrying a functional *p1* gene accumulate flavan-4-ols in the pericarp; flavan-4-ols later polymerize into condensed phlobaphenes, phenotypically seen as red kernels. On the other hand, maize plants carrying a non-functional *p1* gene show no pericarp pigmentation and thus kernel appear yellow or white depending on the color of the endosperm [13,14,15]. Biochemical analyses have shown that phlobaphenes-containing maize lines possess higher antioxidant capacity than conventional lines, a promising trait that could be introduced into elite cultivars to increase dietary benefits [16,17].

Although a large number of studies have illuminated the association between consumption of foods rich in flavonoids and improvement in IBD symptoms, it is difficult to pinpoint the efficacy of any specific flavonoid compound within a whole food matrix. Near-isogenic lines (NILs) are powerful tools to address the beneficial effects from a single class of compounds against colonic inflammation. Such populations are developed through repeated backcrossing of introgression lines to their recurrent parent and contain only a small number of genomic fragments from the donor parent while maintaining a homogeneous genetic background [18]. To specifically address the health benefits of phlobaphenes, we utilized two maize NILs *P1-rr* (F+) and *p1-ww* (F−) that differ only in the content of flavan-4-ols as supplements to feed mice under CMC exposure. We herein report that F+ supplemented diet ameliorated CMC-induced adiposity and low-grade colonic inflammation, as evidenced by reduced epididymal fat-pad weight, lessened pro-inflammatory mediators production and enhanced intestinal barrier function.

## 2. Materials and Methods

### 2.1. Chemicals

Sodium carboxymethylcellulose (Mw~250,000) was purchased from Sigma (St. Louis, Missouri, USA). All other chemicals were also purchased from Sigma unless otherwise mentioned.

### 2.2. Maize Near-Isogenic Lines

Maize near-isogenic lines were named after different pericarp and cob-glumes pigmentation patterns: *P1-rr* (functional *p1* allele, red pericarp, red cob glumes) and *p1-ww* (non-functional *p1* allele, white pericarp, white cob glumes). The *p1-ww* (4Co63) inbred line was obtained from the National Seed Storage Laboratory (Fort Collins, CO, USA) and *P1-rr4B2* genetic stock was obtained from Dr. Thomas Peterson (Iowa State University, Ames, IA, USA). At Penn State University, the *P1-rr4B2* genetic stock was further backcrossed into 4Co63 genetic background. Near-isogenic lines, *P1-rr* (4Co63), and *p1-ww* (4Co63) were grown at Penn State Agronomy Farm, Rock Springs, PA during summers of 2015 for sample collections used in this study.

### 2.3. Maize Sample Extraction

Sample extraction and protein removal were performed by Bligh–Dyer method [19]. In summary, 600 µL methanol containing 0.1% formic acid (*v*/*v*) was added to 30 mg maize sample, vortex and sonicated, then 540 µL water containing 0.1% formic acid (*v*/*v*) and chloroform was added. Samples were shaken for 5 min, followed by centrifugation at 5000 ×g for 30 min. The upper phase was transferred into a new microcentrifuge tube and stored at −20 °C until further use.

### 2.4. Determination of Total Phenolic Content

The total phenolic characterization of maize extracts was determined using the Folin–Ciocalteu (FC) method. A detailed methodology for the procedure has been described previously [20]. The absorbance was read at 765 nm on a Cytation microplate reader (BioTek, Winooski, VT, USA). The values are expressed as milligrams of gallic acid (GA) equivalents per 100 g of corn sample (mg GA equivalents/100 g dry weight).

### 2.5. Total Antioxidant Activity

The antioxidant potential of the samples was determined using 2,2-Diphenyl-1-picrylhydrazyl (DPPH) assay [21]. Briefly, a 24 percent solution of DPPH was made in ethanol, which was further diluted with ethanol until the spectrophotometer (BioTek, Winooski, VT, USA) read 1.1 at 515 nm. A fixed volume of this diluted DPPH mixture 285 μL was pipetted into 15 μL sample, and the reaction was allowed to continue for 2 h. The absorbance of this mixture was then monitored using spectrophotometry at 515 nm. The radical scavenging activity was compared to a standard curve of known dilutions of Trolox. Analyses were run in triplicates and are expressed as milligrams of Trolox equivalents per 100 g of maize kernel sample (mg Trolox/100 g dry weight).

### 2.6. Flavan-4-ols Quantification from Maize Tissue

Dry kernels were ground and 1 mL 30% HCl/70% butanol (*v*/*v*) was added to powdered tissue in an Eppendorf tube and incubated for 1 h at 37 °C. Samples were then centrifuged at 18,000 ×g for 20 sec. Supernatants were pipetted into Cytation microplate reader (BioTek) and sample absorbance was measured at 565 nm and flavan-4-ols content is reported as absorbance/g kernel used.

### 2.7. Metabolomics Sample Preparation and Extraction 

Maize extract from Bligh–Dyer extraction method was evaporated to dryness in a vacuum concentrator (Eppendorf, Hauppauge, NY, USA). The dried polar fraction was reconstituted in 50 µL of a diluent composed of 95% water and 5% acetonitrile, containing 0.1% formic acid. Reconstituted samples were sonicated for 5 min, centrifugation at 16,000 ×*g* for 8 min, and the supernatants were transferred to HPLC autosampler vials.

### 2.8. HPLC-MS Analysis 

Separations were performed on an Agilent 1290 system (Agilent, Palo Alto, CA, USA), with a mobile phase flow rate of 0.45 mL/min. The metabolites were assayed using a Waters HSS T3 column (1.8 µm, 2.1 × 100 mm), where mobile phases A and B were 0.1% formic acid in ddH_2_O and acetonitrile, respectively. Initial conditions were 100:0 A:B, held for 1 min, followed by a linear gradient to 20:80 at 16 min, then 5:95 at 22.5 min. Column re-equilibration was performed by returning to 100:0 A:B at 23.5 min and holding until 28.5 min. The mass analysis was obtained using an Agilent 6545 Q-TOF mass spectrometer (Agilent, Palo Alto, CA, USA) with ESI capillary voltage +3.5 kV, nitrogen gas temperature 325 °C, drying gas flow rate 8.0 L/min, nebulizer gas pressure 30 psi, fragmentor voltage 130 V, skimmer 45 V, and OCT RF 750 V. Mass data (from *m*/*z* 70-1000) were collected at 3 spectra/sec using Agilent MassHunter acquisition software (v.B.06). Mass accuracy was improved by infusing Agilent Reference Mass Correction Solution (G1969-85001). MS/MS was performed in a Data-dependent Acquisition mode. Peak deconvolution, integration, and alignment were performed using Agilent ProFinder (v.B.08). Bioinformatics was performed using MetaboAnalyst 4.0 (McGill University, Montreal, QC, Canada). Peak areas were normalized by converting to log2 and mean centered. Significance analysis was performed by ANOVA with Tukey posthoc. Metabolites with *p* < 0.001 and fold change > 2 were considered significant. Peak annotations were performed using the Plant Metabolic Network (plantcyc.org) metabolite database, with a mass error of less than 30 ppm.

### 2.9. Mice Experimental Diet Preparation

The nutritional composition of *P1-rr* (F+) and *p1-ww* (F−) was determined by Dairy One Inc. (Ithaca, NY, USA) in accordance with AOAC methods for ash, moisture, dietary fiber, protein and total fat (Table 1). Nutritional analysis data was used to formulate diets (Envigo, Indianapolis, IN, USA), namely, 15% and 25% flavan-4-ols-containing F+ supplemented diet (F+(15)-TD.150396, F+(25)-TD. 150397), 15% flavan-4-ols-lacking F− supplemented diet (F−(15)-TD. 150398) and control diet (TD. 130852). Diet composition is included in Table 2.

### 2.10. Animal Study

Wild Type C57BL/6 mice (*n* = 40; males; 4 weeks old) were purchased from Jackson Laboratories (Bar Harbor, ME, USA), and housed in the animal facility at Penn State University, under institutionally approved protocols (Institutional Animal Care and Use Committee No. 45521). Animals were housed in a controlled environment (12-h daylight cycle, lights off at 18:00) with food and water ad libitum. The animals were weaned at 4 weeks of age on control diet, acclimatized for 1 week, and exposed to a 1% dilution of CMC (*w*/*v*) or vehicle (water) for 15 weeks. After 6 weeks, the mice on CMC-water treatment were exposed to either F+(15) diet, F+(25) diet, F−(15) diet or continued on control diet for an additional 9 weeks. Bodyweight and food and water intake were determined once every week. Fecal samples were collected weekly. Blood and urine samples were collected at the end of weeks 1, 6 and 15. After 15 weeks of CMC-feeding, the animals were euthanized in carbon dioxide chambers and sacrificed by cardiac puncture. Organs were collected for downstream analysis. Weights of colon, liver and adipose, as well as colon length were recorded before freezing. Serum was processed from the blood by allowing the blood to clot and then centrifuged at 5000× g for 15 min. The collected serum was stored at −80 °C until further analysis.

### 2.11. Fecal Flagellin and Serum MPO Quantification

Fecal samples were resuspended in phosphate-buffered saline (PBS) to a concentration of 100 mg and then homogenized for 5 min using a bullet blender (Next Advance, Averill Park, NY, USA) without the addition of beads, followed by 2 min centrifugation at 8000× *g*. Reporter cell suspension was made with HEK-BLUE-mTLR5 cells and HEK-BLUE detection media (Invivogen, San Diego, CA, USA) and applied to fecal homogenates according to manufacturer’s instructions. Purified flagellin (Enzo Life Sciences, Farmingdale, NY, USA) isolated from Salmonella typhimurium was used as an experimental standard. After 24 h of incubation, alkaline phosphatase activity was measured via spectrophotometry absorbance at 635 nm. Serum MPO levels were assayed using Duoset mouse myeloperoxidase ELISA kit (R&D Systems, Minneapolis, MN, USA).

### 2.12. Gene expression Analysis of Inflammatory Marker

Total RNA was extracted from colonic tissue with PureLink RNA Mini kit (ThermoFisher Scientific, Hampton, NH, USA), and subjected to qScript cDNA SuperMix (Quantabio, Beverly, MA, USA) for reverse transcription with established protocol. Quantitative real-time PCR (qRT-PCR) was performed using SYBR Green FastMix (Quantabio) in LightCycler 96 (Roche, San Francisco, CA, USA) and the following gene-specific primers (Integrated DNA Technologies, Coralville, IA, USA): IL-6 forward: 5′-AGTTGCCTTCTTGGGACTGA-3′; reverse: 5′-CAGAATTGCCATTGCACAAC-3′; TNF-α forward: 5′-GAACTGGCAGAAG-AGGCACT-3′; reverse: 5′-AGGGTCTGGGCCATAGAACT-3′; TLR4 forward: 5′-ATGGCATGGCTTACACCACC-3′; reverse: 5′-GAGGCCAATTTTGTCTCCA-CA-3′; NFκB forward: 5′-ATGGCAGACGATGATCCCTAC-3′; reverse: 5′-TGTTGACAGTGGTATTTCTGGTG-’. Results were normalized to endogenous control mouse β-Actin (forward: 5′-GGCTGTATTCCCCTCCATCG-3′; reverse: 5′-CCAGTTGGTAACAATGCCATGT-3′).

### 2.13. Tissue Histology

Distal colon tissues were fixed and paraffin-embedded using Leica TP1020 (Leica Biosystems, Buffalo Grove, IL, USA) paraffin processor and Leica EG1150G paraffin embedding unit (Microscopy and Cytometry Facility, Pennsylvania State University). The embedded tissue samples were sectioned and stained with hematoxylin and eosin (H&E) by Animal Diagnostic Laboratory (Pennsylvania State University) prior to histopathology analysis. An experienced blinded evaluator scored all colonic samples based on mucosal damage (IMD), inflammatory cell infiltration severity (ICI-S), inflammatory cell infiltration extent (ICI-E), and submucosal edema using a “0 to 4” scheme. For IMD, 0—within normal limits; 3—crypts are nearly gone, remnants of crypts and the epithelial layer remain; For ICI-S, 0—within normal limits; 4—marked >51%; For ICI-E, 0 - within normal limits; 3—mucosal, submucosal and transmural infiltrates; For edema, 0—none; 3—severe. Results are expressed as accumulative scores of the four parameters.

### 2.14. Intestinal Permeability Assay

Following a 4 h fasting period, mice were orally gavaged with 600 mg/kg body weight of permeability tracer Fluorescein isothiocyanate (FITC)-labelled dextran 4 kDa. Blood was collected from the submandibular site after 3 h, and fluorescence intensity was determined in the serum (λex = 490 nm; λem = 520 nm) using a CLARIOstar Microplate Reader (BMG Labtech, Cary, NC, USA). A standard curve was obtained by performing serial dilutions of FITC dextran in mice serum.

### 2.15. Mucus Thickness Assay

To determine the thickness of the colonic mucus layer, Chassaing’s protocol was followed with minor modifications [4]. Proximal colon tissue was fixed in methanol-Carnoy’s solution (60% *v*/*v* methanol, 30% *v/v* chloroform, 10% *v/v* glacial acetic acid) at room temperature for 10 days. The fixed tissue was washed two times in methanol for 30 min, two washes in ethanol for 15 min, and then once again in an ethanol/xylene solution (1:1 *v*/*v*) for 15 min. Finally, the tissue was washed in xylene two times for 15 min, paraffin-embedded in a vertical orientation, and sectioned at 5 µm thickness before mounting on positively charged slides. The slides were dewaxed by placing in an oven at 50 °C for 30 min. The sections were washed three times in CitriSolv for 5 min, two times in 100% ethanol for 5 min, two times in 95% ethanol for 3 min, once in 70% ethanol for 3 min and then finally rinsed in distilled water for 5 min. The slides were then washed in wash buffer for 10 min, and then three times in PBS for 10 min. A PAP pen (Vector Laboratories, Burlingame, CA, USA) was used to mark the boundaries around the section, and block solution was applied for 30 min at 4 °C. Then, the sections were incubated at 4 °C with Mucin-2 primary antibody (rabbit H-300, Santa Cruz Biotechnology, Dallas, TX, USA), diluted to 1:1500 in blocking solution. The slides were washed three times with PBS for 10 min and incubated with goat anti-rabbit Alexa 488 secondary antibody (Fisher Scientific, Waltham, MA, USA) in block solution (1:1500 dilution) for 2 h. This was followed by three washes in PBS for 10 min, and then the sections were mounted using VECTASHIELD hardset antifade mounting media with DAPI (Vector Laboratories, Burlingame, CA, USA). Mucus thickness was measured using fluorescent microscopy (Olympus BX-61, Melville, NY, USA).

### 2.16. Statistical Analysis

Data are presented as means ± SEM. Statistical analysis was performed using Students’ *t*-test or one-way analysis of variance (ANOVA) corrected for multiple comparisons post hoc Tukey analysis via RStudio v0.99.903 (Boston, MA, USA). All results were considered statistically significant when *p* < 0.05.

## 3. Results

### 3.1. Flavan-4-ols Accumulation Differs in Maize Near-Isogenic Lines P1-rr and p1-ww and Possesses Greater Antioxidant Activity

**Figure 1:**
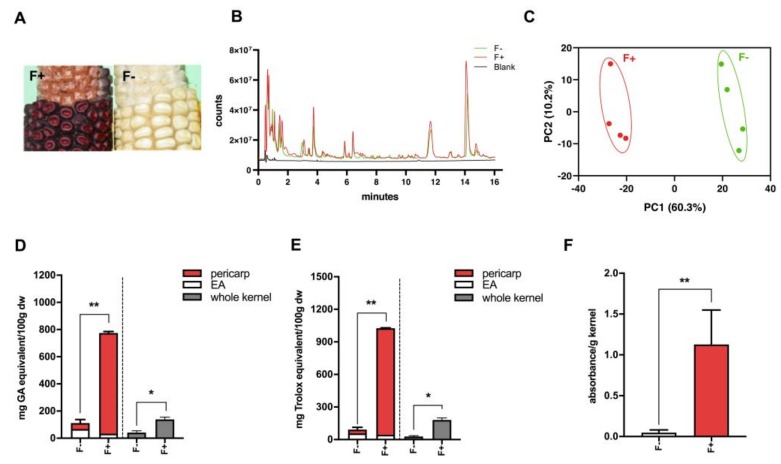
(**A**) Cob phenotype showing kernel pigmentation. Left panel shows red *P1-rr* (F+) corn, and right panel shows white *p1-ww* (F−) corn. F+ accumulates flavan-4-ols displayed by red-colored kernels whereas F− lacks color pigmentation; (**B**) Global metabolomics overlay chromatogram of F+ and F− methanol extracted fragments; (**C**) Principal coordinates analysis of the Euclidean distance matrix of F+ and F− methanol extracted fragments; (**D**) Total phenolic content of F+ and F− based on gallic acid standards assayed with Folin–Ciocalteu reagent; (**E**) Antioxidant activity of F+ and F− based on Trolox standards assayed with 2,2-Diphenyl-1-picrylhydrazyl (DPPH); (**F**) Quantification of flavan-4-ols in F− and F+. EA (endosperm with aleurone layer). * *p* < 0.05, ** *p* < 0.01, two-tailed Student’s *t*-test.

Maize flavan-4-ols biosynthesis requires a transcription factor *pericarp color 1 (p1)*, which is a Myb-related gene that regulates accumulation of flavan-4-ols. A condensed form of flavan-4-ols is red-pigmented phlobaphenes. Two maize near-isogenic lines (NILs) used in this study were: P1-rr, which carried functional *p1* allele and accumulated flavan-4-ols, showing a dark red color on the kernels; p1-ww, which harbored non-functional *p1* allele, conversely, showed no pigmentation (Figure 1A). Acidified butanol (HCl:butanol = 3:7) was used as a solvent for flavan-4-ol extraction and quantification, as flavan-4-ols are unstable under acidic condition and can quickly releases red flavylium ions that are heat-labile and have a λmax around 565 nm [13]. In order to build up the metabolite profile for F+ and F−, we conducted both methanol and acid butanol extraction. Methanol extracts were used for non-targeted metabolomics profiling whereas acid butanol extracts were used for flavan-4-ols quantification. Peak alignment of F+ and F− resulted in 1399 identified peaks, among which 27 compounds were identified as unique to F+, whereas 15 compounds were unique to F−. Since flavan-4-ols were not extracted with methanol, no major peak differences were observed in the overlay chromatogram of F+ and F− (Figure 1B). Principle coordinates analysis (PCA) results suggested a differentiated metabolite profile between F+ and F− (Figure 1C). Biochemical analyses showed that high accumulating flavan-4-ols in F+ pericarp were the predominant phenolic compounds and endowed F+ with significantly higher antioxidant activity compared to F− (Figure 1D–F).

### 3.2. Flavan-4-ols Enriched Maize Diet Alleviates CMC-Induced Adiposity in Wild-Type Mice

**Figure 2:**
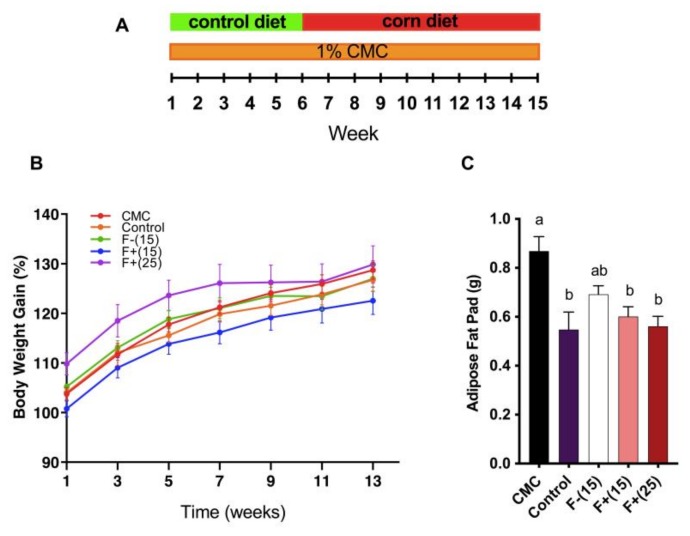
(**A**) Animal experiment scheme. Control group mice were fed with control diet the whole time without carboxymethylcellulose (CMC) exposure. (**B**) Mice body weight gain over time. Body weight is shown as percentage gain compared to week 0; (**C**) Epididymal fat-pad weight. Mice were exposed to CMC in drinking water (1%) for 6 weeks, after that mice were fed with diets supplemented with 15% F+ (F+(15)) or 25% F+ (F+(25)) or 15% F− (F−(15)) or continued on control diet for 9 weeks. The control group mice were fed with a control diet for 15 weeks. Significance level (*p* < 0.05) determined by one-way ANOVA followed by Tukey’s post-test. Bars assigned to different letters are significantly different.

Adiposity is one of the metabolic syndrome manifestations and associated with stronger pro-inflammatory potential and a higher risk of cardiovascular diseases [22]. Previous studies have shown that CMC intake as low as 0.1% resulted in obvious weight gain in WT mice while 1% CMC intake resulted in increased fat mass and fasting blood glucose [4]. In our study, administration of CMC to 5-week old WT mice resulted in elevated fat mass, although a smaller difference was found in terms of overall body weight gain. Alternatively, we found that F+(15) diet and F+(25) diet greatly reduced mice adiposity fat-pad levels, indicating the positive effect of flavan-4-ol on counteracting dietary emulsifier-induced adiposity (Figure 2A, B).

### 3.3. Flavan-4-ols Enriched Maize Diet Alleviates CMC-Induced Low-Grade Intestinal Inflammation in Wild-Type Mice

**Figure 3:**
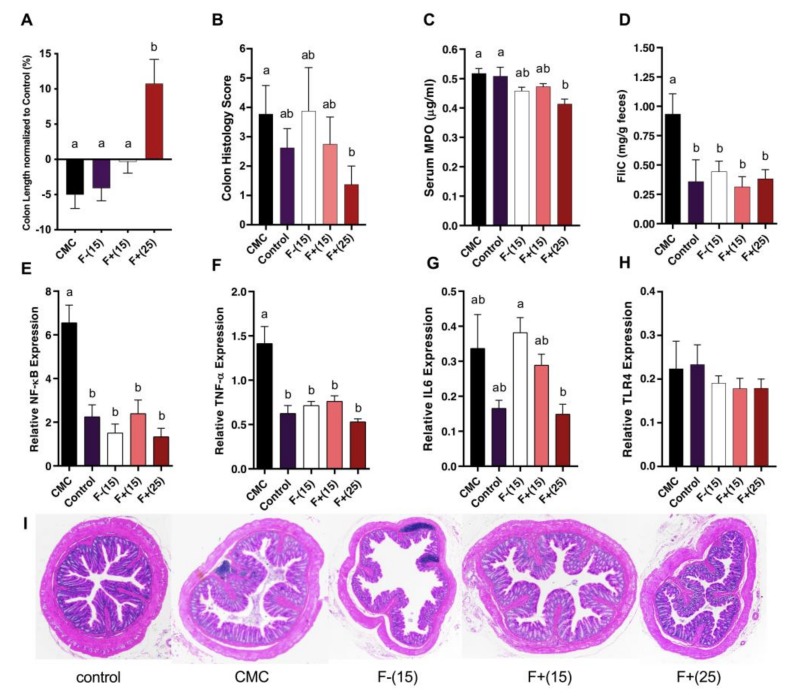
(**A**) Colon length of CMC, F−(15), F+(15) and F+(25) fed mice normalized to control diet fed mice; (**B)** Cumulative histopathology scores for distal colon tissue; (**C**) Serum myeloperoxidase (MPO) levels; (**D**) Bioactive fecal flagellin (FliC) levels assayed with mTLR5 reporter cells. Analysis of relative mRNA expression level of four inflammatory markers NF-κB (**E**), TNF-α (**F**), IL6 (**G**), and TLR4 (**H**) in colonic tissue by qRT-PCR. (**I**) Representative hematoxylin and eosin (H&E)-stained distal colon sections. Significance level (*p* < 0.05) determined by one-way ANOVA followed by Tukey’s post-test. Bars assigned different letters are significantly different.

CMC treated mice exhibited various inflammation features, however, these features were greatly alleviated under F+ treatment. Compared to those on the control diet, CMC fed mice tended to have shorter colons, a common histological feature found in patients with IBD and thought to be linked with crypt erosion. On the other hand, F+(15) fed mice had similar colon length compared to the control group while F+(25) fed mice had around 20% longer colon than CMC treated mice (Figure 3A). To quantify the inflammation levels for colonic tissue, H&E-stained slides were scored based on levels of epithelial damage and inflammatory cell infiltration in the mucosa. In the case of CMC, no significant mucosal damage or inflammatory infiltrates were found under the histopathological examination (Figure 3B). To further investigate the colonic inflammation triggered by CMC as well as the efficacy of F+ supplementation against this inflammation, we utilized several reliable IBD biomarkers including heme enzyme myeloperoxidase (MPO), fecal flagellin (FliC) and pro-inflammatory genes Nuclear factor-kappa B (NF-κB), Tumor necrosis factor α (TNF-α), Interleukin 6 (IL6), and Toll-like receptor 4 (TLR4). Mice fed F+(25) diet showed decreased serum MPO level, which could not be found in any other treatments (Figure 3C). Both F+ diets were able to mitigate fecal FliC, NF-κB, and TNF-α levels whereas F+(25) seemed to be more effective in suppressing the expression of IL6, indicating a less active status of the host immune signaling under F+(25) diet. The expression levels of TLR4 showed no significant differences under all diets including CMC (Figure 3D-H). Together, these data corroborate that flavan-4-ols-containing diets moderated CMC-induced low-grade intestinal inflammation.

### 3.4. Flavan-4-ols Enriched Maize Diet Restores Intestinal Mucosal Barrier Function in CMC-Treated Wild-Type Mice

**Figure 4:**
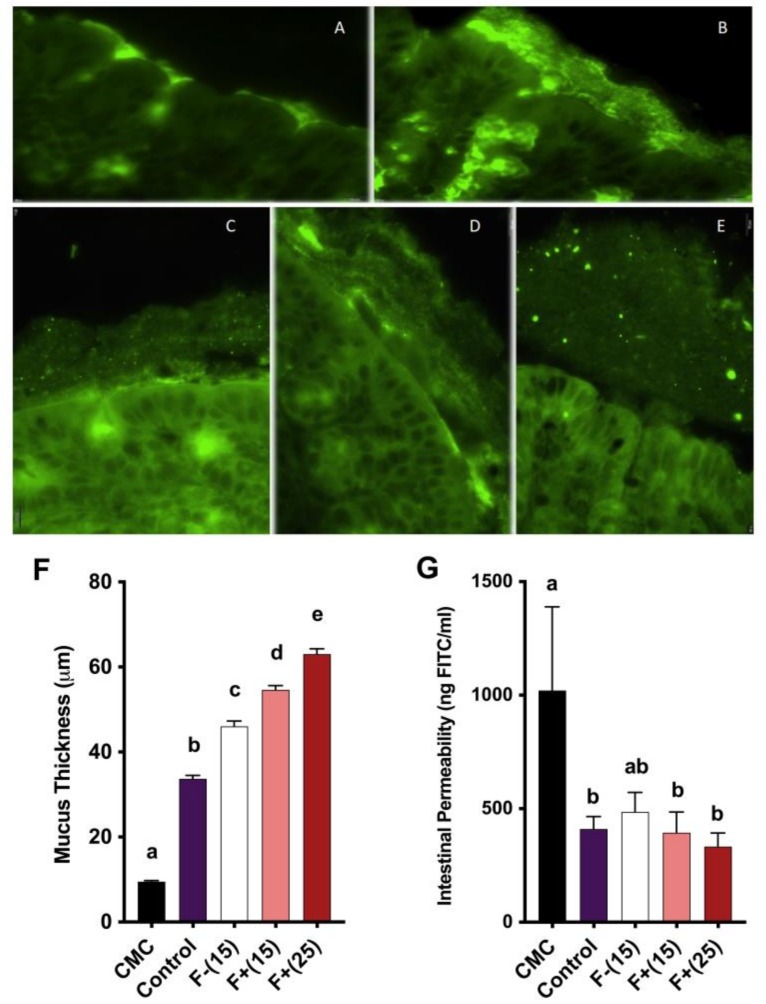
(**A**)–(**E**) Fluorescent microscopy analysis of mice colon mucus thickness stained for MUC2 (green); (**A**) CMC; (**B**) Control; (**C**) F−(15); (**D**) F+(15); (**E**), F+(25). Scale bar: 10 μm at 60X magnification. (**F**) Quantification of mice mucus thickness, results are representative of at least 250 measurements per treatment; (**G**) Mice intestinal permeability assayed by serum FITC-dextran levels 3 h post-gavage. Significance level (*p* < 0.05) determined by one-way ANOVA followed by Tukey’s post-test. Bars assigned to different letters are significantly different.

Intestinal mucus barrier integrity is essential for gut homeostasis and has important antimicrobial properties. Impaired mucosal barrier function is characterized by a thinned mucus layer, increased gut permeability and elevated infiltration of leukocytes and pro-inflammatory bacteria. Our representative colon tissue section images demonstrated gross attenuation of the mucus layer under CMC treatment and amelioration of this attenuation under both F+(15) and F+(25) treatment, indicating a positive role played by flavan-4-ols to restore intestinal barrier function in the context of colonic inflammation (Figure 4A-F). We also tested mucus barrier integrity by measuring the amount of fluorescent-labeled FITC-dextran passing through the intestinal barrier. Serum collected from F+(15) and F+(25) fed mice contained less FITC-dextran compared to CMC treated mice (Figure 4G), which corresponded to increased barrier function integrity.

## 4. Discussion

The past decade has witnessed a worldwide increase in the incidence of IBD. The US alone has 3.1 million IBD patients, suffering from the impaired quality of life caused by this chronic gastrointestinal disorder [23]. Despite being extensively studied, IBD etiology remains elusive, making it challenging for researchers to look for a stable and effective remedy.

Plant bioactive compounds are attracting more attention as protective agents against chronic diseases due to their ubiquity, safety and great anti-inflammatory potential. The food matrix greatly influences nutrient absorption and digestion, thereby altering the bioavailability of flavonoids lie within. Flavonoids in fruits and vegetables are in the soluble free form whereas flavonoids in cereal grains are prevalently in the insoluble bound form. Insoluble bound phenolics in maize make up 85% of the total phenolics. Recent studies have revealed that compared to free soluble phenolics, bound phenolics displayed significantly higher antioxidant capacity and had a longer duration of their presence in the plasma [24,25,26,27]. With the help of maize NILs, our study is able to investigate the beneficial property of a specific class of flavonoid compound flavan-4-ol against colonic inflammation within a whole-food matrix. Our results showed that nutrient compositions of F+ and F− were similar, though the presence of phlobaphene was the major difference between the two NILs, their metabolite profiles were also quite different. This result can be explained by a previous study where researchers reported that genome-wide expression analyses on *P1-rr* and *p1-ww* revealed 5565 differentially expressed gene models and 1586 putative P1 target genes, suggesting that a wide range of genes were subject to P1 regulation [28]. 

Chassaing et al. [4] reported that exposure to CMC altered microbiota ecology and promoted colitis and metabolic syndrome in wild-type mice, supported by findings such as increased body weight, blood glucose and bioactive flagellin level. Herein we report similar findings. Upon CMC exposure, we found that F+ treatment considerably decreased epididymal fat-pads, demonstrating its potential against CMC-induced adiposity. 

Aberrant gut microbiota triggers host innate immune response and results in greater pro-inflammatory potential. MPO is actively involved in reactive oxygen species (ROS)-mediated antimicrobial defenses against IBD and its enzymatic activity is positively associated with inflammation severity [29]. Flagellin as the building block of bacterial filament can bind to host Toll-like receptor 5 (TLR5) and trigger downstream immune signaling and pro-inflammatory cytokine production [30]. Thus we measured mice serum MPO and FliC levels respectively to quantify immune system activation. We found that intake of F+ was associated with decreased serum MPO and FliC levels. Gene expression of pro-inflammatory cytokines NF-κB, TNF-α and IL-6 were reduced with F+ supplementation. Collectively these results support the positive effects of flavan-4-ols intake against CMC-induced low-grade colonic inflammation. 

These conclusions were also confirmed by the observation of restored intestinal mucus barrier function and the enhanced intestinal permeability we observed under F+ treatment. The glycoprotein-rich mucus layer enables the mechanical response of the large intestine to external force and serves as the first line of defense against invading bacteria by preventing direct contact from the bacteria to the host epithelium [31]. Compromised mucus barrier function is associated with a wide range of chronic diseases as it allows for the translocation of pathogenic components into the bowel wall and leads to immune system activation. Previously published literature has reported that flavonoid compounds such as naringenin treatment prevented intestinal barrier function defects in colitic mice [32]. In this experiment, we show that flavan-4-ols containing corn remarkably restored epithelial barrier function under CMC exposure via mucus layer protection and reduced barrier permeability. Among all the measurements, F+(25) tended to have overall a better performance than F+(15), indicating that this beneficial effect is dose-dependent. This lines up with the findings of similar studies [12,33]. The less notable differences between F+(15) and F+(25) are likely due to the low-grade inflammation induced by CMC, further studies using acute DSS colitis model should be conducted to clarify this. 

Plants produce over 6500 flavonoid compounds and a variety of other beneficial components [34]. When considering the complexity of plant food, it is highly likely that bioactive compounds work synergistically to optimize their anti-inflammatory potential, thus contribution made by single health-promoting compound may be less obvious. Flavonoid biosynthetic pathways in maize have been extensively studied, among which flavan-4-ols and flavan-3,4-diols pathways are well identified. The former pathway gives rise to reddish-brown phlobaphenes while the latter gives rise to purple anthocyanins [13,15]. The strong implication of anthocyanins playing an effective antioxidant role, in addition to well-characterized anthocyanin biosynthetic pathway in maize, makes it rational to breed for NIL that accumulates both flavan-4-ols and flavan-3,4-diols to study the possible synergism between the two flavonoid compounds. 

In summary, our data show that flavan-4-ols enriched diet ameliorates CMC-induced colonic inflammation on a whole-food basis. These results may provide a reference for the utilization of these compounds as nutraceuticals. Future work on the health benefits of flavan-4-ols or other bioactive compounds will further public acknowledgment on colored maize cultivar and inspire related product development in the long term. 

## Figures and Tables

**Table 1:** Analyzed nutrient contents of *P1-rr* (F+) and *p1-ww* (F−).

	F−	F+
Nutrient analysis (%)		
Moisture	7.2	8.6
Dry Matter	92.8	91.4
Crude Protein	12.6	15.7
Crude Fiber	2.6	2.1
Crude Fat	3.3	3.4
Ash	1.3	1.4

**Table 2:** Composition of experimental mice diets.

	Control	F+(15)	F+(25)	F−(15)
Ingredient (g/kg)				
Casein	200	172.9	155	178.2
Corn starch	392.23	275.18	197.18	271.98
Soybean oil	70	65.2	61.8	65.2
Cellulose	50	48.95	48.25	46.85
White corn	na	na	na	150
Red corn	na	150	250	na
Nutrient analysis (% by wt)				
Protein	17.7	17.7	17.7	17.7
Carbohydrates	60.1	60.1	60.1	60.3
Fat	7.2	7.2	7.2	7.2
Kcal/g	3.8	3.8	3.8	3.8

na – not applicable.
